# Modification Effects of B_2_O_3_ on The Structure and Catalytic Activity of WO_3_-UiO-66 Catalyst

**DOI:** 10.3390/nano8100781

**Published:** 2018-09-30

**Authors:** Xinli Yang, Nan Wu, Yongxia Miao, Haobo Li

**Affiliations:** College of Chemistry, Chemical and Environmental Engineering, Henan University of Technology, Zhengzhou 450001, China; wunan199204@hotmail.com (N.W.); renamiao@hotmail.com (Y.M.); lihaobo_9@hotmail.com (H.L.)

**Keywords:** UiO-66, WO_3_, B_2_O_3_, metal organic frameworks, cyclopentene, glutaraldehyde

## Abstract

Tungsten oxide (WO_3_) and boron oxide (B_2_O_3_) were irreversibly encapsulated into the nanocages of the Zr-based metal organic framework UiO-66, affording a hybrid material B_2_O_3_-WO_3_/UiO-66 by a simple microwave-assisted deposition method. The novel B_2_O_3_-WO_3_/UiO-66 material was systematically characterized by X-ray diffraction, Fourier transform infrared spectroscopy, N_2_ adsorption, ultraviolet–visible diffuse reflectance spectroscopy, scanning electron microscopy, transmission electron microscopy, X-ray phosphorescence, and Fourier transform infrared (FTIR)-CO adsorption methods. It was found that WO_3_ and B_2_O_3_ were highly dispersed in the nanocages of UiO-66, and the morphology and crystal structure of UiO-66 were well preserved. The B_2_O_3_ species are wrapped by WO_3_ species, thus increasing the polymeric degree of the WO_3_ species, which are mainly located in low-condensed oligomeric environments. Moreover, when compared with WO_3_/UiO-66, the B_2_O_3_-WO_3_/UiO-66 material has a little weaker acidity, which decreased by 10% upon the B_2_O_3_ introduction. The as-obtained novel material exhibits higher catalytic performance in the cyclopentene selective oxidation to glutaraldehyde than WO_3_/UiO-66. The high catalytic performance was attributed to a proper amount of B_2_O_3_ and WO_3_ with an appropriate acidity, their high dispersion, and the synergistic effects between them. In addition, these oxide species hardly leached in the reaction solution, endowing the catalyst with a good stability. The catalyst could be used for six reaction cycles without an obvious loss of catalytic activity.

## 1. Introduction

WO_3_ based catalysts have been widely used in many chemical reactions, such as hydrocarbon isomerization [1,2], dehydration and cracking [3,4], oxidation [5,6], etherification and alkylation [7,8], selective reduction of nitric oxide with ammonia [9,10], and photocatalytic reactions, owing to their various catalytic properties [11,12]. The surface area of the pristine WO_3_ is low and the homogeneous WO_3_ catalyst is difficult to recycle. Loaded WO_3_ catalyst on various solid carriers could improve the physicochemical properties and can be conveniently separated and recovered. Previous studies indicate that the nature of the solid carrier strongly affects the structure of WO_3_, its dispersion, oxidation state, and surface acidity. Accordingly, all of these factors significantly affect the catalytic performance of WO_3_.

Among the tungsten oxide based catalysts, WO_3_/ZrO_2_ catalyst, first reported as a strongly acidic catalytic system by Hino and Arata [13], has been widely explored in acid-catalyzed reactions. Despite their high activity and thermal stability, WO_3_/ZrO_2_ catalysts have the disadvantage of a low surface area and non-uniform pore size, limiting their applications for catalyzing larger bulky molecules. Then, mesoporous silicas, such as MCM-41 and SBA-15, were introduced into the system of tungsten oxide-zirconia catalyst. Jiménez-López et al. synthesized a series of Zr-doped MCM-41 supported WO_3_ catalysts by the impregnation method and studied their catalytic performance for the esterification of oleic acid with methanol [14]. Ge and coworkers prepared a high surface area WZr/SBA-15 catalyst by the hydrothermal method for the hydrolysis of cellobiose, with significantly higher activity than a conventional WO_3_/ZrO_2_ catalyst that was prepared by the impregnation method [15]. In addition to zirconia and mesoporous silica materials, tungsten oxide has also been supported on other metal oxides, such as titania, tin oxide, and ceria. Supported WO_3_/TiO_2_ catalysts possess both strongly acidic and modest redox characteristics and have been mainly used for the selective catalytic reduction (SCR) of NO*_x_* with NH_3_. Recently, Wachs et al. prepared WO_3_/TiO_2_ by the co-precipitation and impregnation methods and investigated the effect of catalyst preparation method on their catalytic activity. The co-precipitated catalyst exhibited slightly enhanced SCR reactivity [16]. So far, tungsten oxide supported on tin oxide materials have been extensively used in acid catalysis. Kamata et al. proved that the heterogeneous catalyst W-Zn/SnO_2_, synthesized by tungsten and zinc oxides that were loaded on SnO_2_ while using a two-step impregnation method, showed high efficiency for the selective oxidation of various sulfides, alkenes, silanes, and amines using aqueous hydrogen peroxide as the green oxidant. Moreover, this catalyst could be recycled several times without any obvious deactivation [17]. Dai et al. found that the catalytic activity of WO_3_/SnO_2_, prepared by the co-precipitation-impregnation method, was strongly affected by the calcination temperature of the catalyst WO_3_/SnO_2_ and the support SnO_2_. The appropriate calcination temperature of the support and catalyst led to the well dispersion of W species, and a few W(VI) ions entered into the SnO_2_ lattice, thus improving the catalytic activity for the selective oxidation of 1,2-benzenedimethanol [6]. Because of its smaller bandgap and better absorption of visible light, WO_3_ has also been extensively used to promote photocatalytic reactions by combination with TiO_2_ or noble metal of Pt. Dong et al. introduced the Pt/WO_3_ composites on various solid carriers (e.g., ceramics, activated carbon, molecular sieves, and alumina) by the sol–gel method and investigated their photocatalytic activity for the removal of NO gas [12]. Gupta and coworkers successfully synthesized MWCNT/WO_3_ catalyst by supporting WO_3_ on multi-walled carbon nanotubes, exhibiting enhanced photocatalytic activity when compared to WO_3_ itself for the decomposition of organic dye under visible light [18].

Hitherto, WO_3_ has been supported on different solid carriers to enhance its catalytic performance. Nevertheless, to the best of our knowledge, supported WO_3_ on metal-organic frameworks (MOFs), a new class of porous crystalline solids, which were formed by metal ions or inorganic clusters coordinated to organic linkers [19,20], is hardly investigated. MOFs, which could be designed by varying organic linkers and inorganic joints, have attracted significant attention in recent years by virtue of their permanent porosity, open crystalline structures, tunable cavities, and extraordinary surface areas. These unique and outstanding characteristics offer a great potential range of applications in separation, gas storage, magnetism, sensor, drug release, membrane technology, and catalysis [21,22,23,24]. For example, Livingston et al. prepared a kind of hybrid polymer/MOF membrane by in-situ growth (ISG) of HKUST-1 within the pores of polyimide membranes. To improve the performances of ISG membranes, chemical modification was performed. They found that chemically modified ISG membranes outperformed non-modified ISG membranes in both solute retentions and permeance [25]. Mohammed et al. reported that platinummetallated porphyrin (Pt(II)TMPyP) was successfully encapsulated in a rho-type zeolite-like metal-organic framework (rho-ZMOF) and applied for anion-selective sensing. The sensing activity and selectivity of the MOF-encaged Pt(II)TMPyP for various anions in aqueous and methanolic media were significantly enhanced when compared to that of the free (non-encapsulated) Pt(II)TMPyP [26]. Farha et al. utilized an ALD-like process (ALD: atomic layer deposition) to obtain the UiO-66-supported nickel catalysts. Moreover, three Ni-decorated UiO-66 materials were synthesized by varying the number of ALD cycles, which exhibited catalytic for ethylene hydrogenation process under mild conditions after thermal activation. A clear activesite size effect on the catalytic activity is observed, with the largest catalyst sites displaying the highest activity [27]. Notably, Lillerud et al. reported the Zr-based MOF in 2008 and synthesized UiO-66 under conventional solvothermal conditions using ZrCl_4_ and 1,4-benzenedicarboxylate (BDC) as the metal precursor and organic ligand, respectively, in *N*,*N*-dimethylformamide (DMF) [28]. UiO-66 consists of Zr_6_O_4_(OH)_4_ octahedral units that were connected by 12-fold linear BDC struts to form a rigid three-dimensional cubic close-packed network, possessing high thermal and mechanical stability and good chemical resistance to various solvents, such as ethanol, benzene, and water, owing to their high affinity of Zr to oxygen ligands and the compact structure. Moreover, the octahedral and tetrahedral cages of 11 and 8 Å attributes high porosity to UiO-66, and thus could be easily accessible through microporous triangular windows in the range 5–7 Å. All of these unique properties are beneficial to application in catalysis and they make UiO-66 a good candidate for encapsulating WO_3_.

In our previous research, WO_3_ supported on the silica-based molecular sieves has been proven to be efficient heterogeneous catalysts for the preparation of glutaraldehyde (GA) by the selective oxidation of cyclopentene (CPE). GA is widely used in the fields of disinfection and sterilization [5,29,30,31]. The detailed studies documented that the large surface area and the microporous or mesoporous characteristics of the siliceous molecular sieves favor catalysts’ high activity for the selective oxidation of CPE. Therefore, WO_3_ encapsulated into the nanocages of UiO-66 is likely to be a suitable catalyst for the selective oxidation of CPE. In general, WO_3_ loaded on different supports is synthesized via two steps. First, WO_3_ precursor is deposited on the functional support by a conventional impregnation method, and then the obtained material is dried and calcined at the appropriate temperature. When compared to the inorganic materials, MOFs have lower thermal stability, and such MOFs with encapsulated WO_3_ could not be prepared by the conventional impregnation and calcination method. Herein, we demonstrate a green and facile microwave-assisted deposition method to prepare a series of WO_3_ and B_2_O_3_ encapsulated in UiO-66 catalysts (B_2_O_3_-WO_3_/UiO-66) with excellent catalytic performance for the selective oxidation of CPE to GA. To the best our knowledge, the present work is the first attempt to utilize MOFs as the supports to prepare WO_3_ based catalysts. Furthermore, the effect of B_2_O_3_ used as an active additive on the catalytic activity and structure of the B_2_O_3_-WO_3_/UiO-66 catalysts was studied. The relationship between the catalytic activity and structure of B_2_O_3_-WO_3_/UiO-66 catalyst was concisely discussed based on N_2_ adsorption, X-ray diffraction (XRD), Fourier transform infrared (FTIR) spectroscopy, scanning electron microscopy (SEM), transmission electron microscopy (TEM), UV–vis diffuse reflectance spectroscopy (DRS), X-ray phosphorescence (XPS), and FTIR-CO adsorption analyses. 15 wt % B_2_O_3_–40 wt % WO_3_/UiO-66 catalyst exhibited optimal catalytic performance for the selective oxidation of CPE, indicating that a certain amount of B_2_O_3_ and WO_3_ and the synergistic effects between them are essential for the good catalytic activity of B_2_O_3_-WO_3_/UiO-66.

## 2. Materials and Methods

### 2.1. Synthesis of Materials

The chemical reagents used in the experiments were as follows: zirconium chloride (ZrCl_4_, 99.9%; aladdin, Shanghai, China), terephthalic acid (H_2_BDC, AR, 98%; Aldrich, Shanghai, China), *N*,*N*-dimethylformamide (HCON(CH_3_)_2_, DMF, AR, 99.8%; aladdin, Shanghai, China), methanol (CH_3_OH, AR, 99.5%; Sinophram Chemical Reagent Co. Ltd. Shanghai, China), ethanol (CH_3_CH_2_OH, AR, 99.7%; Sinophram Chemical Reagent Co. Ltd, Shanghai, China), Tert-butyl alcohol ((CH_3_)_3_OH, AR, 99.9%; aladdin, Shanghai, China), concentrated HCl (CP, 37%; The Third Company of Tianjin Chemical Reagents, Tianjin, China), tungstic acid (WO_3_·H_2_O, CP, 99%; The Third Company of Tianjin Chemical Reagents, Tianjin, China), hydrogen peroxide solution (H_2_O_2_, AR, 50 wt %; The Third Company of Tianjin Chemical Reagents, Tianjin, China), cyclopentene (CPE, AR, 99%; Aldrich, Shanghai, China), and cyclopentane ( AR, 99%; Aldrich, Shanghai, China). 

All of the materials were supplied from commercial sources and were directly used without any treatment.

#### 2.1.1. Synthesis of UiO-66

UiO-66 was prepared based on the hydrothermal procedure reported in the literature [32]. 1.45 g ZrCl_4_, 1.06 g terephthalic acid (H_2_BDC), and 0.5 mL concentrated HCl were dissolved in 40 mL DMF. Then, the mixture was sealed in a 100 mL Teflon-lined stainless steel autoclave and crystallized at 120 °C for 24 h. The mixture was further cooled naturally to room temperature, separated by centrifugation, and washed by centrifuging with DMF several times to remove excess H_2_BDC. Then, the obtained white powder was washed with methanol and dried at 180 °C for 10 h. The yield of UiO-66 based on zirconium is ca. 68%. 

#### 2.1.2. Synthesis of WO_3_/UiO-66

WO_3_/UiO-66 (WO_3_ encapsulated in UiO-66) was prepared by a simple microwave-assisted deposition method. The preparation of the catalyst is as follows: A certain amount of tungstic acid, WO_3_·H_2_O, was added to hydrogen peroxide (50%) to obtain the oxoperoxo-tungstate sources. The molar ratio of WO_3_·H_2_O:H_2_O_2_ was approximately 1:50. After the mixture was stirred at 60 °C for an hour, a transparent tungsten complex-containing solution was obtained. Pure UiO-66 was added into the above solution and continued to stir for further 6 h under the same conditions. Then, the mixture was heated under microwave at 150 W for 15 min in a CEM Discover microwave reactor (average temperature 80 °C, 100 °C maximum temperature reached). Finally, the white solid was separated from the solution by vacuum filtration, washed with deionized water and ethanol, and then dried overnight in the air at 180 °C for 10 h.

#### 2.1.3. Synthesis of B_2_O_3_-WO_3_/UiO-66

The encapsulated B_2_O_3_ and WO_3_ in UiO-66 catalyst, denoted as B_2_O_3_-WO_3_/UiO-66, was synthesized by the same simple microwave-assisted deposition method. A certain amount of tungstic acid (WO_3_·H_2_O) and boric acid (H_3_BO_3_) was added to hydrogen peroxide (50%) to obtain the oxoperoxo-species sources. Then, the followed process is the same as that of WO_3_/UiO-66.

In order to ensure the reproducibility of the synthesis for UiO-66, WO_3_/UiO-66, and B_2_O_3_-WO_3_/UiO-66, the synthesis of all the materials were repeated at least three times. The XRD, FTIR, and SEM results show that all the materials could be successfully prepared. Additionally, in order to increase the sustainability of the UiO-66 and B_2_O_3_-WO_3_/UiO-66 synthesis, the toxic solvents such as DMF and hydrogen peroxide should be minimized and be sustainably recovered using membranes which have been reported by Szekely et al. [33,34].

### 2.2. Characterizations of Materials

The crystalline phases of the as-prepared samples were characterized by XRD (Rigaku D/max-rB, Tokyo, Japan) equipped with Cu Kα radiation under 60 mA and 40 kV. FTIR measurements were collected using a Shimadzu-IR Prestige-21 spectrometer (KyoTo, Japan) with KBr pellets. In situ FTIR spectra were recorded using a Bruker-Tensor 27 spectrophotometer (Karlsruhe, Germany) with a DTGS detector using CO as the probe molecule. The BET surface area and pore volume were measured by N_2_ adsorption isotherms using a Micromeritics ASAP 2020 instrument (Atlanta, GA, USA) at −196 °C. Scanning electron micrographs were obtained using a JEOL scanning electron microscope (JSM-6510LV, Tokyo, Japan). TEM images were recorded while using a Philips transmission electron microscope (Tecnai F20, Hillsborough, OR, USA). Thermogravimetric experiments were performed in air atmosphere (50 mL·min^−1^) using a Perkin Elmer TGA7/DTA7 system (Waltham, MA, USA). UV–visible diffuse reflectance spectra were acquired using a UV–vis spectrometer (UV-2540, Shimadzu, KyoTo, Japan) with BaSO_4_ as the reference standard. XPS spectra were analyzed using X-ray photoelectron spectroscopy (Perkin-Elmer PHI 5000C ESCA, Waltham, MA, USA) equipped with a hemispheric energy analyzer. A Mg Kα anode was used as the radiation. All of the binding energies were corrected with contaminant C 1s (284.6 eV) as the reference. The WO_3_ and B_2_O_3_ contents were detected by inductively coupled plasma (ICP, Thermo ICP-OES 6500, Waltham, MA, USA).

### 2.3. Test of Catalytic activity

The catalytic activity of the materials was tested by the selective oxidation of CPE. All the reactants and a certain amount of catalyst were put in a sealed 100 mL regular glass reactor and magnetically stirred at 308 K for 24 h. The products were analyzed by GC and different products in the reaction mixture were identified by GC-MS. Details can be found elsewhere [35,36].

All the tests were repeated at least three times, and the experimental errors were within (5%).

## 3. Results

### 3.1. Catalyst Characterizations

**Figure 1:**
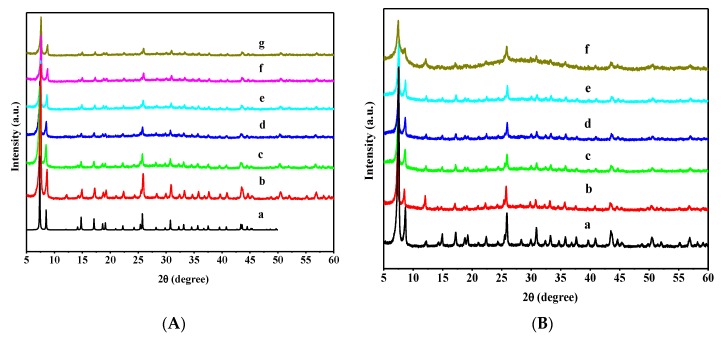
(**A**) X-ray diffraction (XRD) patterns of various samples: (a) UiO-66, reproduced with permission from [37]. American Chemical Society, 2011; (b) UiO-66; (c) 20 wt %; (d) 30 wt %; (e) 35 wt %; (f) 40 wt %; (g) 45 wt % WO_3_/UiO-66; (**B**) XRD patterns of various samples: (a) UiO-66; (b) 5 wt % B_2_O_3_-40 wt % WO_3_; (c) 10 wt % B_2_O_3_-40 wt % WO_3_; (d) 15 wt % B_2_O_3_-40 wt % WO_3_; (e) 20 wt % B_2_O_3_-40 wt % WO_3_/UiO-66; and, (f) 15 wt % B_2_O_3_-40 wt % WO_3_ after the sixth reaction cycle.

The crystalline phases of the obtained materials were measured by powder XRD, and the results are shown in Figure 1. When compared to the standard powder XRD pattern of UiO-66 derived from the CIF-file [37], the powder XRD pattern of the pristine UiO-66 matches well with the simulated one, confirming the as-synthesized material as UiO-66 with pure crystalline phase (Figure 1A). The intensity of peaks attributed to WO_3_/UiO-66 catalysts decreased with increasing WO_3_ contents; however, the XRD patterns of a series of WO_3_/UiO-66 catalysts hardly exhibit any difference with the pristine UiO-66, demonstrating that the crystal of UiO-66 maintained its structure after WO_3_ encapsulation in UiO-66, and it is in accordance with the results that were reported by Férey et al. and Gascon et al. [38,39]. Moreover, compared to the pristine UiO-66, the 2θ of WO_3_/UiO-66 catalysts shifted to a lower diffraction angle, suggesting that introducing WO_3_ into the nanocages of UiO-66 possibly increased the distance of the adjacent lattice planes of UiO-66. This result is consistent with the results reported by Lu et al. [40], where the phosphotungstic acid in MIL-101 increased the distance between the adjacent lattice planes. Furthermore, even when the WO_3_ content increased to 45 wt % in the WO_3_/UiO-66 catalysts, WO_3_ could not be detected in the XRD patterns of the catalysts, indicating the UiO-66 support has larger surface area and higher porosity, thus leading to the even dispersion of WO_3_. Figure 1B exhibits the XRD patterns of different B_2_O_3_-WO_3_/UiO-66 catalysts. No distinct loss of crystallinity was observed, and the basic lattice structure of UiO-66 remains unchanged upon the encapsulation of B_2_O_3_ in the WO_3_/UiO-66 catalyst, indicating that WO_3_/UiO-66 itself is not significantly affected by B_2_O_3_. In addition, characteristic XRD patterns of B_2_O_3_ were not observed, demonstrating the uniform distribution of B_2_O_3_ in the B_2_O_3_-WO_3_/UiO-66 catalyst. Figure 1B also presents the XRD pattern of 15 wt % B_2_O_3_-40 wt % WO_3_/UiO-66 catalyst after three successive reaction cycles, and the XRD pattern is nearly identical to that of the freshly prepared one, proving high stability of UiO-66 material in the reactive system.

**Figure 2:**
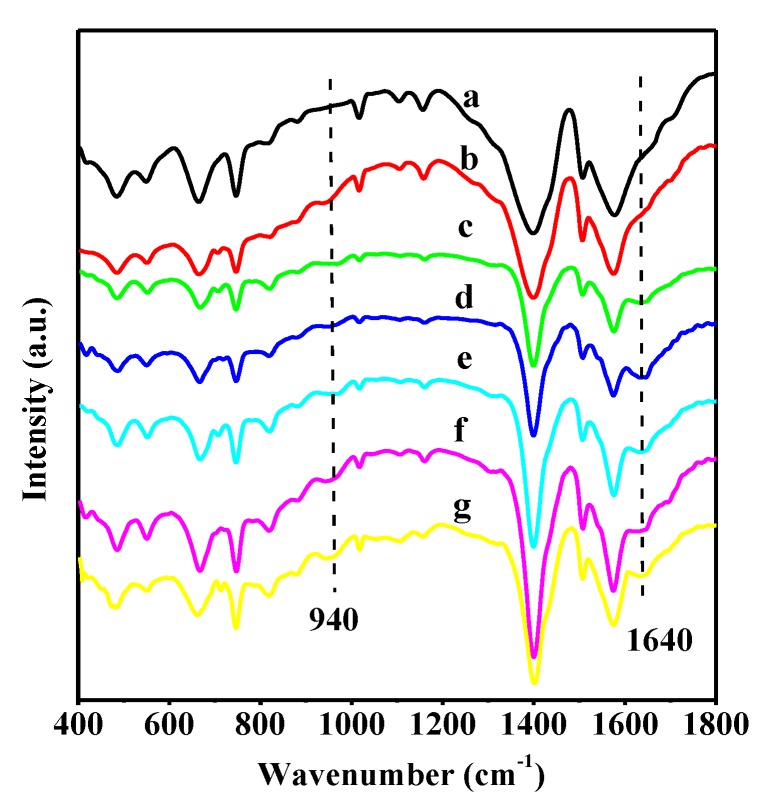
Fourier transform infrared (FTIR) spectra of various samples: (a) UiO-66; (b) 40 wt % WO_3_/UiO-66; (c) 5 wt % B_2_O_3_-40 wt % WO_3_; (d) 10 wt % B_2_O_3_-40 wt % WO_3_; (e) 15 wt % B_2_O_3_-40 wt % WO_3_; (f) 20 wt % B_2_O_3_-40 wt % WO_3_/UiO-66; and, (g) 15 wt % B_2_O_3_-40 wt % WO_3_ after the sixth reaction cycle.

**Figure 3:**
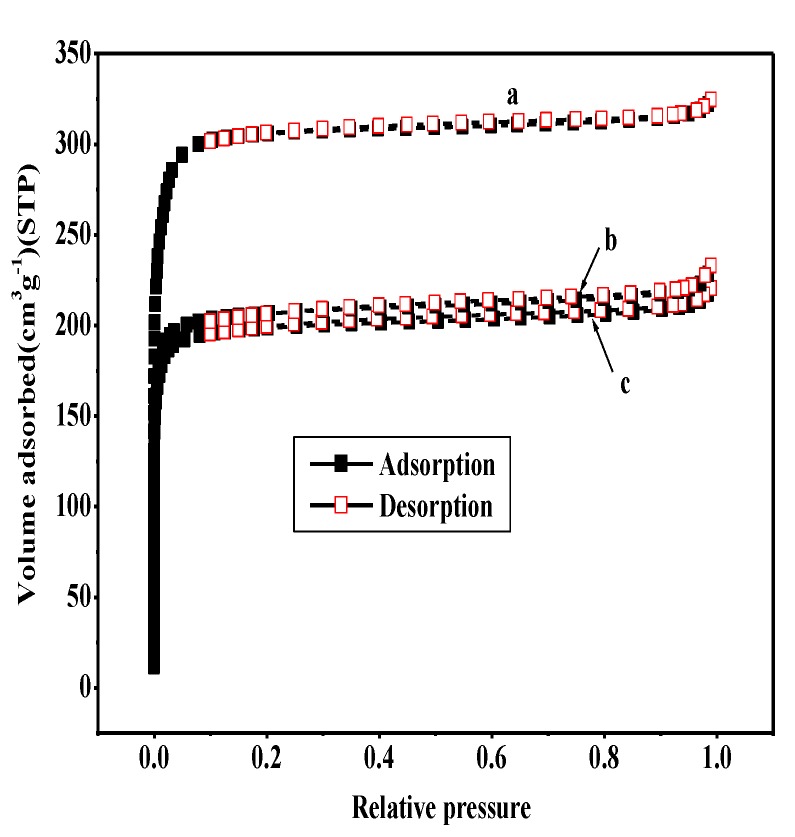
Nitrogen sorption isotherms for (a) UiO-66; (b) 40 wt % WO_3_/UiO-66; and, (c) 15 wt % B_2_O_3_-40 wt % WO_3_/UiO-66.

The incorporation of B_2_O_3_ and WO_3_ species into the UiO-66 support was investigated by FTIR, and the results indicate that the structure of UiO-66 is preserved in the WO_3_/UiO-66 and B_2_O_3_-WO_3_/UiO-66 samples (Figure 2). As shown in the spectrum of the pristine UiO-66, the sharp intensity peak at 1400 cm^−1^ is attributed to the stretching vibrations of C=C bound in the aromatic ring. The strong bands in the region 1800–1300 cm^−1^ and the weak ones in the range 700–400 cm^−1^ are assigned to the different stretching and bending vibrations of the COO groups, respectively, indicating the presence of H_2_BDC linker in the UiO-66 framework. Especially, the band at 550 cm^−1^ was assigned to the Zr–O vibrations, confirming the formation of UiO-66 MOFs [41]. The spectra of the 40 wt % WO_3_/UiO-66 samples show the characteristic peak of WO*_x_* species at 940 cm^−1^, as well as the characteristic infrared bands corresponding to UiO-66 (Fig. 3b) [16]. For different B_2_O_3_-WO_3_/UiO-66 samples, a new band appears at 1640 cm^−1^, related to the B–O stretching vibration of B_2_O_3_ according to the previous report that the bands in the range 1200–1600 cm^−1^ correspond to the stretching vibrations of the BO_3_ groups [42]. The FTIR results indicate that WO_3_ and B_2_O_3_ were introduced in the UiO-66 support by the microwave-assisted deposition method. Moreover, the FTIR spectrum of 15 wt % B_2_O_3_-40 wt % WO_3_/UiO-66 remains unchanged after the third catalytic cycle, indicating that the catalyst has a highly stable structure, and this result is in accordance with the XRD result.

Table 1 shows the BET and Langmuir surface area, pore diameter, and total pore volume of the different samples. The permanent porosity of the samples was confirmed by the N_2_ physisorption method (Figure 3). At a low relative pressure, the strong N_2_ uptake of all the samples manifests their microporous characteristic [43,44]. UiO-66 has a Langmuir surface area, a BET surface area, and a pore volume of 1344 m^2^·g^−1^, 1062 m^2^·g^−1^, and 0.46 cm^3^·g^−1^, respectively, exhibiting the type I isotherms in the N_2_ adsorption isotherms at −196 °C with no hysteresis loop. In the case of UiO-66, the BET surface area is very close to that reported in the literature [28] (1069 m^2^·g^−1^), demonstrating that UiO-66 could be easily synthesized. When compared to the pristine UiO-66 support, the Langmuir and BET surface areas, and the pore volumes of 40 wt % WO_3_/UiO-66 catalysts obviously decreased. Although the N_2_ adsorption isotherm maintains the type I shape, the volume adsorbed by N_2_ clearly decreased after the incorporation of WO_3_. All of the evidence indicates that WO_3_ species occupied the micropores of the UiO-66 material, thus decreasing the surface area and pore volume. The surface area and pore volume of 15 wt % B_2_O_3_-40 wt % WO_3_/UiO-66 sample were found to be similar to that of 40 wt % WO_3_/UiO-66 sample, expected from the strong interaction between B_2_O_3_ and WO_3_ species and the enwrapped action of WO_3_ species for B_2_O_3_ species. 

**Figure 4:**
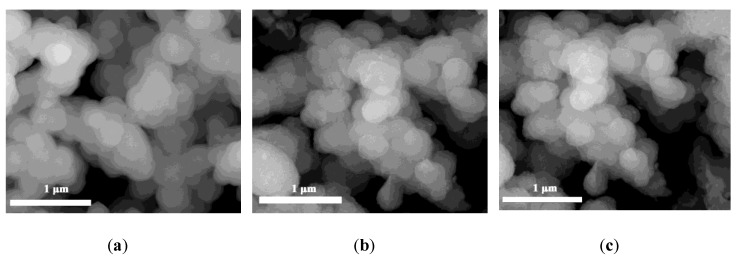

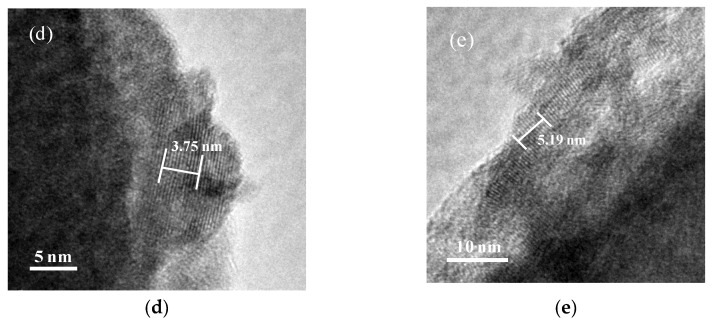
Scanning electron microscopy/transmission electron microscopy (SEM/TEM) images of various samples. SEM images of (**a**) UiO-66; (**b**) 40 wt % WO_3_/UiO-66; (**c**) 15 wt % B_2_O_3_-40 wt % WO_3_/UiO-66; High-resolution TEM images of (**d**) UiO-66; and, (**e**) 15 wt % B_2_O_3_-40 wt % WO_3_/UiO-66.

The SEM and TEM images of UiO-66, 40 wt % WO_3_/UiO-66, and 15 wt % B_2_O_3_-40 wt % samples are shown in Figure 4. The SEM images indicate that all the three samples comprised agglomerated uniform small particles, presenting the characteristic irregular inter-grown microcrystalline poly-octahedra morphology. Figure 4d and Figure 4e are the HRTEM images of UiO-66 and 15 wt % B_2_O_3_-40 wt % samples. As shown in Figure 4d, the average distance between the adjacent lattice plane of UiO-66 is 0.375 nm calculated from the 10 lattice plane distances, whereas it is 0.519 nm for the 15 wt % B_2_O_3_-40 wt % WO_3_/UiO-66 sample (Figure 4e) larger than that of UiO-66, and is expected to be induced by the introduction of WO_3_ and B_2_O_3_ [40,45]. This corroborates well with the XRD studies and confirms that WO_3_ and B_2_O_3_ have been successfully incorporated into the nanocages of UiO-66 by the microwave-assisted deposition method. The SEM and TEM images confirm the incorporation of WO_3_ and B_2_O_3_ in UiO-66, without changing the morphology and structure of UiO-66. 

**Figure 5:**
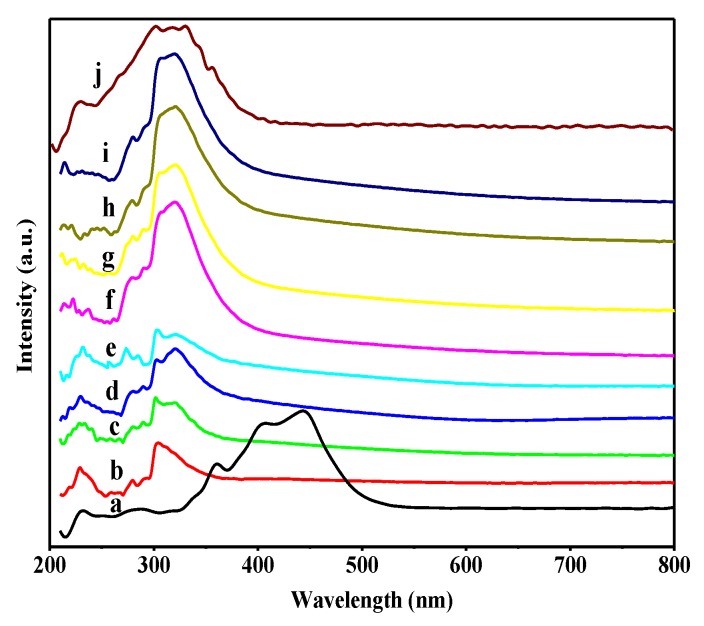
UV-Vis diffuse reflectance spectra of various samples. (a) Bulk Tungsten oxide (WO_3_); (b) 20 wt %;(c) 30 wt %; (d) 35 wt %; (e) 40 wt % WO_3_/UiO-66; (f) 5 wt % B_2_O_3_-40 wt % WO_3_; (g) 10 wt % B_2_O_3_-40 wt % WO_3_; (h) 15 wt % B_2_O_3_-40 wt % WO_3_; (i) 20 wt % B_2_O_3_-40 wt % WO_3_/UiO-66; and, (j) 11.4 wt % W-HMS.

The speciation of WO_3_ species and the effect of B_2_O_3_ on the coordination states of WO_3_ species were surveyed by the UV–vis DRS technique, and the results are shown in Figure 5. All of the spectra were obtained by deducting the spectrum of the support UiO-66. For comparison, the UV–vis DRS spectrum of bulk WO_3_ is also presented, exhibiting a strong absorption band at 450 nm with a weak shoulder at 360 nm, accompanied by relatively weaker bands at 230 and 280 nm (Figure 5a), and these bands are attributed to crystalline WO_3_ [5,46], isolated WO_4_ tetrahedral species [47], and isolated or low condensed oligomeric tungsten oxide species in octahedral coordination [48], respectively. When compared to bulk WO_3_, different WO_3_/UiO-66 samples absorbed at 230, 280, and 310 nm (Figure 5b–e), which was ascribed to the absorption of isolated or low condensed oligomeric tungsten oxide species. Although the strong peak at 310 nm shifts to slightly higher wavelength side with increasing amounts of WO_3_, no peaks at 450 nm appeared in these samples, suggesting that there is no crystalline WO_3_, and the tungsten oxide species are well dispersed in the nanocages of UiO-66. As for the series of B_2_O_3_-WO_3_/UiO-66 samples (Figure 5f–i), all of the peaks at 230, 280, and 310 nm shifted to a higher wavelength; however, no absorption peak at 450 nm corresponding to crystalline WO_3_ was observed. Moreover, the intensity of the peak at 240 nm decreased with increasing amounts of doped B_2_O_3_, whereas the intensity of the bands at 320 nm increased and the bands become dominant. Weber et al. reported that the UV–vis DRS spectra of tungsten oxide or molybdenum oxide species would shift to a lower wavelength side with decreasing polymeric degree of tungsten or molybdenum entity [49,50]. Therefore, the doping of B_2_O_3_ and the interaction of B_2_O_3_ and WO_3_ prevent the formation of isolated tungsten oxide species; however, it leads to the agglomeration of tungsten oxide species. In addition, most of the tungsten oxide species in the B_2_O_3_-WO_3_/UiO-66 samples exist in a low condensed oligomeric form, while a small fraction exists as monotungstate species. Despite the fact that the polymeric degree of tungsten oxide species increases, crystalline WO_3_ was not observed, and the B_2_O_3_-WO_3_/UiO-66 catalysts with the appropriate amount of B_2_O_3_ exhibits high catalytic activity for the selective oxidation of CPE to GA.

**Figure 6:**
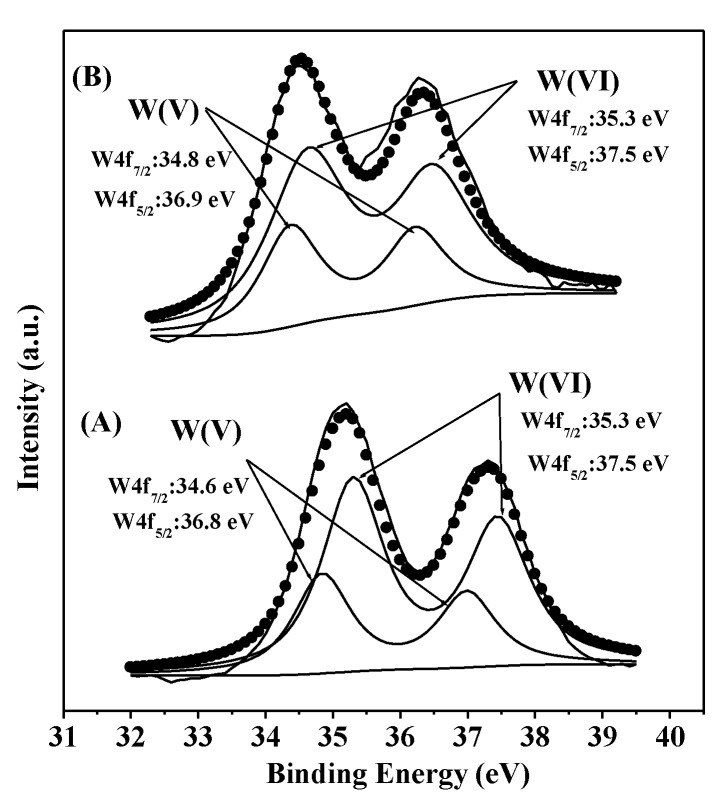
X-ray phosphorescence (XPS) spectra of the W4f region for (**A**) 40 wt % WO_3_/UiO-66 and (**B**) 15 wt % B_2_O_3_-40 wt % WO_3_/UiO-66.

XPS measurement was used to investigate the chemical state of the tungsten and boron species in 40 wt % WO_3_/UiO-66 and 15 wt % B_2_O_3_-40 wt % WO_3_/UiO-66 samples. Figure 6A shows the W 4f XPS spectrum and the curve-fitting results of 40 wt % WO_3_/UiO-66 sample. Tungsten species appear in two different states in the 40 wt % WO_3_/UiO-66 material. The peaks with the binding energies at 35.3 and 37.5 eV are associated with W^5+^ species, whereas the peaks at 34.6 and 36.8 eV can be assigned to the W^6+^ species [51]. The corresponding results for 15 wt % B_2_O_3_-40 wt % WO_3_/UiO-66 sample are shown in Figure 6B. The XPS spectrum of 15 wt % B_2_O_3_-40 wt % WO_3_/UiO-66 material appears to be same as that of 40 wt % WO_3_/UiO-66 material and it shows identical positions for the W 4f peaks, except for the minor charging effect. Both W^5+^ and W^6+^ species are detected; however, the molar ratio of W^6+^/W^5+^ calculated according to the relative peak intensity of W 4f increases from 2.1 to 2.2 (Table 1) after the introduction of B_2_O_3_ species as an active additive promoter, suggesting that the content of W^6+^ species on the surface of B_2_O_3_-WO_3_/UiO-66 sample increases and forms some aggregated tungsten oxide species [5]. The result is probably attributed to the strong interaction between tungsten oxide and boron oxide, and the relatively high electronic affinity of boron oxide, decreasing the degree of oxidation of W^5+^ to W^6+^ species. The same phenomenon has also been reported by Yuan and coworkers [52]. They found that boron oxide doped in Cu-SiO_2_ catalyst decreased the reduction of surface copper and caused a partial positive charge on the copper surfaces, because of the electronic affinity and the stronger interaction with cupreous species of boron oxide. Interestingly, the XPS spectrum of 15 wt % B_2_O_3_-40 wt % WO_3_/UiO-66 sample did not show the presence of boron species; however, the FTIR band appearing at 1640 cm^−1^ is related to the vibration of B_2_O_3_. In addition, the ICP analysis also shows the presence of B_2_O_3_, indicating that the B_2_O_3_ species are not distributed on the outer surface of 15 wt % B_2_O_3_-40 wt % WO_3_/UiO-66 sample, thus indicating that B_2_O_3_ species are enwrapped in WO_3_ species, and this result is consistent with the N_2_ physisorption analysis and the UV–vis DRS results. Although the aggregation degree of tungsten oxides and W^6+^ species increases, no crystalline tungsten oxides are formed, as supported by the XRD and UV–vis DRS results, suggesting that tungsten oxide in 15 wt % B_2_O_3_-40 wt % WO_3_/UiO-66 sample are highly dispersed and increase the catalytic activity for the selective oxidation of CPE to GA.

**Figure 7:**
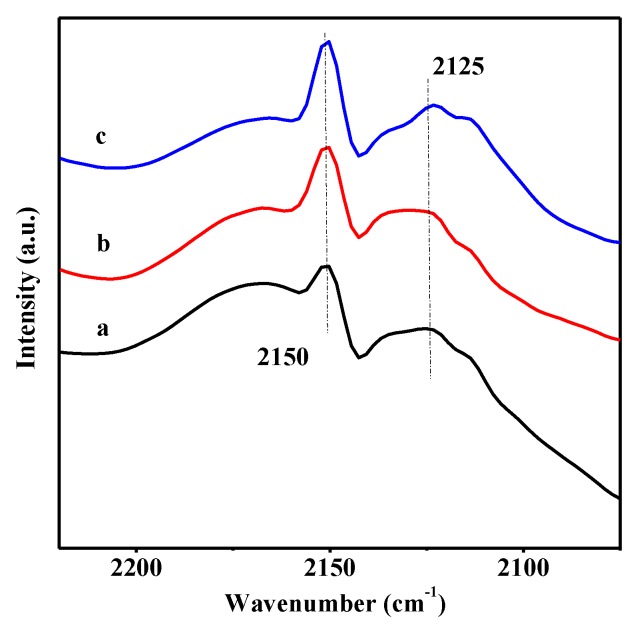
FTIR spectra of various samples recorded after outgassing at 473 K for two hours, after adsorption of CO at room temperature. (a) UiO-66; (b) 40 wt % WO_3_/UiO-66; (c) 15 wt % B_2_O_3_-40 wt % WO_3_/UiO-66.

The properties of the acidic sites in UiO-66, 40 wt % WO_3_/UiO-66, and 15 wt % B_2_O_3_-40 wt % WO_3_/UiO-66 were investigated by in situ infrared spectroscopy using CO as the probe molecule. Figure 7 exhibits the CO-FTIR spectra of the samples that were obtained at liquid nitrogen temperature. Two main bands are present in the UiO-66 spectrum: the first broad peak with a weak shoulder at 2113 cm^−1^ is centered at 2125 cm^−1^ and it is attributed to the physisorbed CO species [43]. The second sharp peak in the UiO-66 spectrum is centered at 2150 cm^−1^, followed by a broad tailed peak at 2170 cm^−1^, which may be ascribed to the CO molecule coordinatively bound up with the heterogenous Lewis acid sites formed by Zr^4+^ in UiO-66 [53]. For 40 wt % WO_3_/UiO-66 material, both of the bands at 2125 and 2150 cm^−1^ increase in intensity, indicating that the acidity of 40 wt % WO_3_/UiO-66 material was obviously enhanced because of the incorporation of tungsten oxides into the support UiO-66. When compared to the 40 wt % WO_3_/UiO-66 material, the intensity of these two bands of 15 wt % B_2_O_3_-40 wt % WO_3_/UiO-66 material becomes slightly weak, which decreased by about 10% through the comparison of peak areas, and might be induced by the addition of boron oxides to 40 wt % WO_3_/UiO-66 material. The XPS and UV–vis DRS results confirm that the strong interaction of boron oxides with tungsten oxides increases the polymeric degree of tungsten oxide species, which in turn, might slightly decrease the acidity of 15 wt % B_2_O_3_-40 wt % WO_3_/UiO-66 material, but it is still stronger than that of UiO-66. The CO probing study reveals that tungsten oxide species in 40 wt % WO_3_/UiO-66 provide additional B and L acid sites, distinct from the Zr^4+^ environment in UiO-66, and boron oxide species attribute 15 wt % B_2_O_3_-40 wt % WO_3_/UiO-66 sample to exhibit appropriate acidity, which is indispensable for the catalytic selective oxidation of CPE to GA.

### 3.2. Catalytic Activity and Stability

The catalytic activities of the WO_3_/UiO-66 catalysts with varying WO_3_ content for the selective oxidation of CPE to GA are listed in Table 2. It is noticed that the original UiO-66 is less active for the selective oxidation of CPE to GA, whereas those with highly dispersed WO_3_ on the UiO-66 support exhibit high activity and selectivity to the reaction. This result discloses that the WO_3_ encapsulated in the nanocages of the UiO-66 provides redox sites that are responsible for the selective oxidation of CPE. Furthermore, the CPE conversion and GA yield increase with increasing WO_3_ content in the WO_3_/UiO-66 catalysts up to 40 wt % WO_3_ loading. Further increasing the WO_3_ loading to 45 wt % slightly decreases the GA yield, probably because of the fact that too much WO_3_ occupies the nanocage of the UiO-66 support and inevitably decreases the surface area and porosity of the material, thus hindering the mass transfer of the reactants to the active centers. This result reveals that the best catalyst, 40 wt % WO_3_/UiO-66, achieved 100% CPE conversion and 64.9% GA yield. However, as compared to our previous studies, the catalytic performance of tungsten oxide supported on UiO-66 (40 wt % WO_3_/UiO-66) is much lower than those that are supported on siliceous mesoporous molecular sieves HMS (Table 2). For 11.4 wt % W-HMS (Si/W = 30) catalyst, the GA yield reaches 76.2% at only 11.4 wt % tungsten oxide loading in the catalysts. The distinct difference in the catalytic activity of the two catalysts may be rationally explained based on the following reasons. First, the physicochemical state of tungsten oxide species in the two materials is different. The UV–vis DRS results indicate that the low-condensed oligomeric tungsten oxide species are predominant in 11.4 wt % W-HMS catalyst, whereas tungsten oxides are present as both isolated tungsten oxide species and low condensed oligomeric tungsten oxide species in UiO-66. The amount of isolated tungsten oxide species in 40 wt % WO_3_/UiO-66 catalyst is higher than that in 11.4 wt % W-HMS catalyst, as supported by the more intense absorption band at 230 nm, attributing the 40 wt % WO_3_/UiO-66 catalyst with higher acidity than 11.4 wt % W-HMS catalyst. The different physicochemical properties of these two supports may be another reason for different catalytic activities of the two catalysts. HMS is an inertia siliceous mesoporous material, with no catalytic activity for the CPE oxidation. UiO-66 is an organic–inorganic hybrid crystalline material with high surface area and permanent micropores, exhibiting a little catalytic activity for the selective oxidation of CPE to GA. As a result, the yield of 2-*t*-butyloxy-1-cyclopentanol (CPLE) over 40 wt % WO_3_/UiO-66 catalyst is two times of than that over 11.4 wt % W-HMS catalyst (Table 2), thus the GA yield is much lower over 40 wt % WO_3_/UiO-66 catalyst because of these two reasons.

Recently, B_2_O_3_ has received special attention as catalyst in many chemical reactions, owing to its suitable acidity. Examples are the partial oxidation of olefins [54,55], the hydrogenation of olefins [51,56], and the hydrogenolysis of glycerol [57]. In this study, B_2_O_3_ was added into 40 wt % WO_3_/UiO-66 catalyst to synthesize a series of B_2_O_3_-WO_3_/UiO-66 catalysts. The catalytic activities of these catalysts for the selective oxidation of CPE to GA were also investigated. As listed in Table 2, the B_2_O_3_-WO_3_/UiO-66 catalyst exhibits higher catalytic performance than the corresponding WO_3_/UiO-66 catalyst. However, the CPE conversion and GA yield increase slowly with increasing B_2_O_3_ loading. It is observed that the highest GA yield reaches 73.4% in the presence of 15 wt % B_2_O_3_-40 wt % WO_3_/UiO-66 catalyst, and further increasing the B_2_O_3_ loading to 20 wt % slightly decreases the GA yield. Although the XRD characterization results indicate that WO_3_ species are highly dispersed in the B_2_O_3_-WO_3_/UiO-66 catalysts, and no crystalline WO_3_ species are formed upon the introduction of B_2_O_3_. The N_2_ adsorption, XPS, and UV–vis DRS measurements reveal that the polymeric degree of tungsten oxide species increases. The N_2_ adsorption results show that 15 wt % B_2_O_3_-40 wt % WO_3_/UiO-66 and 40 wt % WO_3_/UiO-66 have nearly the same surface area and pore volume. Moreover, the XPS characterization indicates that boron species in 15 wt % B_2_O_3_-40 wt % WO_3_/UiO-66 catalyst cannot be detected, thus it can be concluded that the B_2_O_3_ species are enwrapped by WO_3_ species in 15 wt % B_2_O_3_-40 wt % WO_3_/UiO-66 catalyst, leading to the aggregation of tungsten oxide species and the low condensed oligomeric tungsten oxide species become dominant with increasing B_2_O_3_ loading, as confirmed by the UV–vis DRS spectra. The proper amount of the low condensed oligomeiric tungsten oxide species is beneficial to the selective oxidation of CPE to GA.

Since Furukawa et al. firstly reported an interesting one-step route for the synthesis of GA by the selective oxidation of CPE in 1987 [58], there have been great efforts to investigate the reaction system to enhance the GA yield, in which the homogeneous or heterogeneous catalysts based on molybdenum or tungsten heteropoly acids and tungsten oxides were mainly employed. The catalytic activity of various catalysts are summarized in Table S1. The GA yield that was reported by Furukawa et al. did not exceed 60% over all the mentioned homogeneous catalysts in a non-aqueous system while using H_2_O_2_ and TBHP as the oxidant and solvent, respectively (Table S1). Then, a high GA yield of ca. 80% was obtained through aqueous H_2_O_2_ oxidation of CPE using homogeneous tungstic acid as an efficient catalyst by our group [59], which could be expected as an effective approach for the GA synthesis. However, the homogeneous catalyst cannot be recycled easily, thus restricting its further application. Hence, more research efforts have been made to design the heterogeneous tungsten heteropoly acid-based or WO_3_-based catalyst. The good catalytic performance of several heterogeneous catalysts, including W-HMS [29], W-MCM-48 [30], W-SBA-15 [5,31], WO_3_/g-C_3_N_4_ [60], and HPWs@UiO-66 [45] has been delineated in Table S1. Recently, Zhang et al. reported the fabrication of a series of HPW ionic liquids and their catalytic performance for the selective oxidation of CPE to GA [61]. Although the HPW ionic liquids showed excellent catalytic activity, the loss of the catalysts is serious. Compared with the above mentioned catalysts, the 15 wt % B_2_O_3_-40 wt % WO_3_/UiO-66 catalyst also exhibited good performance for this oxidation reaction and the GA yield could reach 73.4%. Therefore, the metal organic framework UiO-66 was suitable for encapsulating WO_3_ and B_2_O_3_ by a facile microwave-assisted deposition method, and then overcome those drawbacks of the homogeneous system.

Apart from having a good catalytic performance, the high stability and the absence of leaching are also important for the heterogeneous catalyst. 15 wt % B_2_O_3_-40 wt % WO_3_/UiO-66 was tested in several consecutive cycles for the selective oxidation of CPE to determine whether the catalyst suffers from the active component leaching and destruction of the porous structure. After each run, the solid was filtered off, washed with ethanol, and dried in the air at 453 K overnight. Then, the catalyst was tested in the next cycle under the same reaction conditions. The possible leaching of the active component was determined by ICP analyses after the reaction completion. As shown in Table 3, 15 wt % B_2_O_3_-40 wt % WO_3_/UiO-66 catalyst can be reused for at least six cycles. No distinct decrease in the catalytic performance of the catalyst in the six cycles could be observed. Notably, there is no distinction between the XRD and FTIR results before and after the reaction, and ICP experiment proved that almost no B and W were detected in the reaction solution, indicating that 15 wt % B_2_O_3_-40 wt % WO_3_/UiO-66 catalyst shows high stability and catalytic activity for the selective oxidation of CPE to GA. We believe that the decrease in the observed catalytic activity is possibly because of the loss of small amounts of catalyst in the process of filtration and washing. Recently, Szekely et al. reported that the covalent grafting of an organocatalyst to a membrane surface would allow for the development of a new, sustainable methodology consisting of catalysis, product purification, excess reagent, and solvent recovery in a single unit operation, in addition to overcoming the lost of catalysts [62]. Pourjavadi el al. prepared a new magnetic MNP@SPGMA@AP@Pd catalyst using a kind of magnetic nanocomposite to immobilize palladium nanoparticles by a simple and ecologically safe method. The prepared catalyst could be magnetically recovered and effectively reused to prevent the loss of catalysts [63]. Hence, it may be an effective method to load B_2_O_3_ and WO_3_ on the membrane or magnetic materials to prevent the catalyst loss, thus avoiding the deterioration of the catalytic performance. Detailed studies is being under way. To establish the heterogeneity of 15 wt % B_2_O_3_-40 wt % WO_3_/UiO-66 catalyst in the reaction, a hot filtering experiment was carried out. After the reaction over 15 wt % B_2_O_3_-40 wt % WO_3_/UiO-66 catalyst was performed for 4 h, the catalyst was collected by simple filtration, and then the reaction solution was stirred for another 20 h. No further reaction was observed in solution, indicating the heterogeneous behavior of the solids.

Based on the characterizations and catalytic activity tests of the catalysts, the 40 wt % WO_3_/UiO-66 catalyst exhibited good catalytic activity for the selective oxidation of CPE because of the highly dispersed tungsten oxide species encapsulated in the nanocages of UiO-66; however, the yield of the side product 2-*t*-butyloxy-1-cyclopentanol (CPLE) is high, probably because too many highly dispersed WO_3_ species exist in the form of isolated species and low condensed oligomeric species with strong acidity in the catalyst. The yield of the target product GA increases, and the yield of CPLE decreases at the optimum amount of B_2_O_3_ species introduced in 40 wt % WO_3_/UiO-66 catalyst. The N_2_ adsorption and XPS analysis demonstrate that the B_2_O_3_ species are wrapped by WO_3_ species and sequentially increases the polymeric degree of WO_3_ species and slightly decreases the acidity of the catalyst, as verified by the UV–vis DRS and CO-FTIR analyses. Therefore, it is conceivable that the synergistic effects of B_2_O_3_ species with WO_3_ species enhance the catalytic activity of 15 wt % B_2_O_3_-40 wt % WO_3_/UiO-66 catalyst for the selective oxidation of CPE to GA, and the strong interactions between the two oxides and the confinement effect of the nanocages of UiO-66 prevent the active components from leaching.

## 4. Conclusions

In summary, novel B_2_O_3_-WO_3_/UiO-66 catalyst was successfully prepared by a simple and eco-friendly microwave-assisted deposition method. The crystalline structure of the UiO-66 framework was well retained upon B_2_O_3_-WO_3_ incorporation. The obtained material as a true heterogenous catalyst showed 100% conversion of CPE and 73.4% selectivity of GA. Moreover, the catalyst exhibited excellent stability for the selective oxidation of CPE to GA, which could be reused for at least six cycles. It was demonstrated that the introduction of B_2_O_3_ species leads to increase in the polymeric degree of WO_3_ species and decrease in the acidity of WO_3_/UiO-66 catalyst confirmed by UV-Vis DRS and CO-FTIR, which enhanced the GA yield from 64.9% to 73.4%. The present study put forward a new, facile, and green method for microwave-assisted deposition synthesis of B_2_O_3_-WO_3_/UiO-66 catalysts, which might open novel vistas for exploring more metal oxide/MOFs materials for green catalytic oxidation applications.

## Figures and Tables

**Table 1:** Physico-chemical parameters of various samples.

Sample	S_Langmuir_ (m^2^·g^−1^)	S_BET_ (m^2^·g^−1^)	Pore Diameter (nm)	Pore Volume (cm^3^·g^−1^)	W^6+^/W^5+^ ^a^
UiO-66	1344	1062	1.9	0.46	-
40 wt %WO_3_/UiO-66	817	684	2.0	0.34	2.1
45 wt % WO_3_/UiO-66	736	592	2.1	0.32	-
15 wt %B_2_O_3_-40 wt %WO_3_/UiO-66	809	677	2.1	0.36	2.2

^a^ Calculated according to the curve-fitting results of the W 4f XPS spectra of catalysts.

**Table 2:** Catalytic performance in the selective oxidation of cyclopentene (CPE) over various samples *^a^*.

Sample (WO_3_/UiO-66 or B_2_O_3_-WO_3_/UiO-66)	WO_3_ Contents wt % *^b^*	B_2_O_3_ Contents wt % *^b^*	Conversion of CPE (%)	GA Yield (%)	Selectivity (%)			
					GA	CPDL	CPLE	Others *^c^*
UiO-66	-	-	21.2	3.8	18.0	13.6	12.3	56.1
20 wt %WO_3_	19.3	-	75.5	24.8	32.9	9.5	32.0	25.6
30 wt %WO_3_	30.7	-	90.5	35.3	39.0	10.1	29.5	21.4
35 wt %WO_3_	34.8	-	96.7	47.0	48.6	12.1	27.1	12.2
40 wt %WO_3_	41.3	-	100	64.9	64.9	13.5	14.2	7.4
45 wt %WO_3_	45.9	-	100	63.8	63.8	12.9	13.6	9.7
5 wt %B_2_O_3_-40 wt %WO_3_	40.8	4.6	100	70.0	70.0	5.8	14.7	9.5
10 wt %B_2_O_3_-40 wt %WO_3_	41.3	9.3	100	71.6	71.6	6.1	13.6	8.7
15 wt %B_2_O_3_-40 wt %WO_3_	40.9	14.5	100	73.4	73.4	4.5	12.1	10.0
20 wt %B_2_O_3_-40 wt %WO_3_	41.1	20.3	100	69.6	69.6	5.2	15.2	10.0
11.4 wt %W-HMS	-	-	100	76.3	76.3	14.6	7.8	1.3

*^a^* Reaction condition: The molar ratio of CPE:H_2_O_2_:WO_3_ = 1:2.5:0.05, the volume ratio of *t*-BuOH/CPE = 10, reaction time 24 h, reaction temperature 308 K; CPE, cyclopentene; GA, glutaraldehyde; CPLE, 2-*t*-butyloxy-1-cyclopentanol; CPDL, cyclpentan-1,2-diol; *^b^* measured by ICP; *^c^* Others, including cyclopentene oxide and cyclopentenone.

**Table 3:** Reusability of 15 wt %B_2_O_3_-40 wt %WO_3_/UiO-66 *^a^*.

Entry	Conversion of CPE (%)	GA Yield (%)	Selectivity (%)			
			GA	CPDL	CPLE	Others
1	100	73.8	73.8	4.3	11.6	10.3
2	100	72.9	72.9	4.1	10.9	12.1
3	98.2	68.9	70.2	4.6	9.8	15.4
4	97.3	67.0	68.9	4.8	9.2	17.1
5	96.8	65.8	68.0	5.9	9.6	16.5
6	96.0	64.7	67.4	5.7	9.8	17.1

*^a^* Reaction condition: The molar ratio of CPE:H_2_O_2_:WO_3_ = 1:2.5:0.05, the volume ratio of *t*-BuOH/CPE = 10, reaction time 24 h, reaction temperature 308 K.

## Supplementary Materials

The following are available online at http://www.mdpi.com/2079-4991/8/10/781/s1, Figure S1: FT-IR spectra of various samples: (a) UiO-66; (b) 40 wt % WO_3_/UiO-66; (c) 5 wt % B_2_O_3_-40 wt % WO_3_; (d) 10 wt % B_2_O_3_-40 wt % WO_3_; (e) 15 wt % B_2_O_3_-40 wt % WO_3_; (f) 20 wt % B_2_O_3_-40 wt % WO_3_/UiO-66; (g) 15 wt % B_2_O_3_-40 wt % WO_3_ after the sixth reaction cycle., Table S1: Comparison of catalytic performance in the selective oxidation of CPE over various samples.
