# Supplementary material for: Modification Effects of B2O3 on The Structure and Catalytic Activity of WO3-UiO-66 Catalyst
Source: Nanomaterials (Basel). 2018 Sep 30;8(10):781. doi: 10.3390/nano8100781 (PMC6215294; doi:10.3390/nano8100781)
Supplement: Supplementary file 1 [file nanomaterials-08-00781-s001.pdf]

Supplementary data

Modification effects of B_2O_3 on the structure and catalytic activity of WO_3-UiO-66 catalyst

Xinli Yang*, Nan Wu, Yongxia Miao and Haobo Li

College of Chemistry, Chemical and Environmental Engineering, Henan University of technology, Henan 450001, P. R. China

*Correspondence: yangxinli_9@126.com (X.Y.); xlyang@haut.edu.cn (X.Y.); Tel.: +86-371-6775-6193

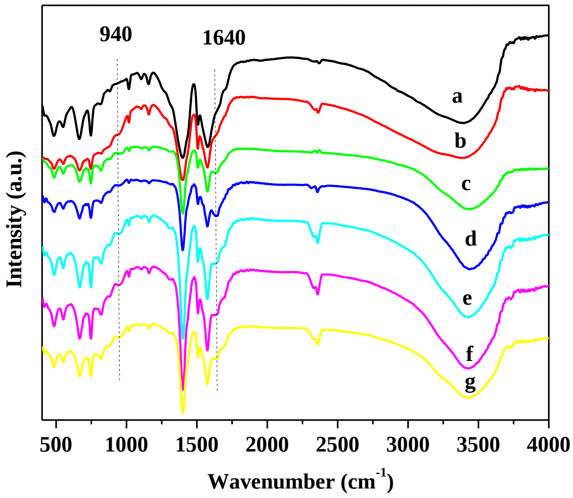

Figure S1. FT-IR spectra of various samples: (a) UiO-66; (b) 40 wt% WO_3/UiO-66; (c) 5 wt% B_2O_3-40 wt% WO_3; (d) 10 wt% B_2O_3-40 wt% WO_3; (e) 15 wt% B_2O_3-40 wt% WO_3; (f) 20 wt% B_2O_3-40 wt% WO_3/UiO-66; (g) 15 wt% B_2O_3-40 wt% WO_3 after the sixth reaction cycle.

Table S1

Comparison of catalytic performance in the selective oxidation of CPE over various samples *

Entry	Sample	Conversion of CPE (%)	GA Yield (%)	Selectivity of GA (%)
1	HPWs[a]	-	5.4	-
2	$H_3PMo_{12}O_{40}$[a]	-	46.5	-
3	$H_3PMo_{10}W_2O_{40}$[a]	-	60.6	-
4	$WO_3 \cdot H_2O$[b]	100	80	80
5	W-HMS[c]	100	76.3	76.3
6	20 wt% W-MCM-48[d]	85.2	66.9	78.5
7	20 wt% WO_3-MCF[e]	100	83.5	83.5
8	20 wt% WO_3-SBA-15[f]	94	78.9	83.9
9	20 wt% WO_3-SBA-15[g]	100	91	91
10	35 wt% HPWs@UiO-66[h]	94.8	78.3	82.6
11	5 wt% WO_3/g-C_3N_4[i]	98.0	75.0	76.5
12	HPWs ionic liquids[j]	100	87.0	87.0

* Entries of 1–4 and 6 are homogeneous catalysts; others are heterogenous catalysts.

[a] Reaction condition: see reference [1]; The catalytic reaction is carried out in an anhydrous H_2O_2/TBP system.

[b] Reaction condition: see reference [2]; $WO_3 \cdot H_2O$ = tungstenic acid.

[c] Reaction condition: see reference [3].

[d] Reaction condition: see reference [4].

[e] Reaction condition: see reference [5].

[f] Reaction condition: see reference [6];

[g] Reaction condition: see reference [7];

[h] Reaction condition: see reference [8].

[i] Reaction condition: see reference [9].

[j] Reaction condition: see reference [10].

[1] Furukawa, H.; Nakamura, T.; Inagaki, H.; Nishikawa, E.; Imai, C. Misong, M. Oxidation of Cyclopentene with Hydrogen Peroxide Catalyzed by 12-Heteropoly Acids. *Chem. Lett.* **1988**, 17, 877–880.

[2] Deng, J.F.; Xu, X.H.; Chen, H.Y.; Jang, A.R. A new process for preparing dialdehydes by catalytic oxidation of cyclic olefins with aqueous hydrogen peroxide. *Tetrahedron*, **1992**, 48, 3503–3514.

[3] Yang, X.L.; Dai, W.L; Chen, H.; Xu, J.H.; Cao, Y.; Li, H.X.; Fan, K.N. Novel tungsten-containing mesoporous HMS material: its synthesis, characterization and catalytic application in the selective oxidation of cyclopentene to glutaraldehyde by aqueous H_2O_2. *Appl. Catal. A Gen.* **2005**, 283, 1–8.

[4] Yang, X.L.; Dai, W.L; Gao, R.H.; Chen, H.; Li, H.X.; Cao, Y.; Fan, K.N. Synthesis, characterization and catalytic application of mesoporous W-MCM-48 for the selective oxidation of cyclopentene to glutaraldehyde. *J. Mol. Catal. A: Chem.* **2005**, 241, 205–214.

[5] Yang, X.L.; Yin, A.Y.; Dai, W.L.; Fan, K.N. Synthesis of Highly Efficient WO3-Doped MCF Catalyst and Its Application in the Selective Oxidation of Cyclopentene to Glutaraldehyde. *Acta Phys.-Chim. Sin.* **2011**, 27, 177-185.

[6] Yang, X.L.; Dai, W.L; Chen, H.; Cao, Y.; Li, H.X.; He, H.Y.; Fan, K.N. Novel efficient and green approach to the synthesis of glutaraldehyde over highly active W-doped SBA-15 catalyst. *J. Catal.* **2005**, 229, 259–263.

[7] Yang, X.L.; Dai, W.L.; Gao, R.H.; Fan, K.N. Characterization and catalytic behavior of highly active tungsten-doped SBA-15 catalyst in the synthesis of glutaraldehyde using an anhydrous approach. *J. Catal.* **2007**, 249, 278–288.

[8] Yang, X.L.; Qiao L.M.; Dai, W.L. Phosphotungstic acid encapsulated in metal-organic framework UiO-66: An effective catalyst for the selective oxidation of cyclopentene to glutaraldehyde. *Micropor. Mesopor. Mater.* **2015**, 211, 73–81.

[9] Ding, J.; Liu, Q.Q.; Zhang, Z.Y.; Liu, X.; Zhao, J.Q.; Cheng, S.B.; Zong, B.N.; Dai, W.L. Carbon nitride nanosheets decorated with WO3 nanorods: Ultrasonic-assisted facile synthesis and catalytic application in the green manufacture of dialdehydes. *Appl. Catal. B Environ.* **2015**, 165, 511–518.

[10] Zhang, J.S.; Yu, F.L.; Tao, R.Q.; Xie, C.X.; Yuan, B.; Yu, S.T. Selective Oxidation of Cyclopentene to Glurarraldehyde Catalyzed by Heteropolyphosphatotungstate Ionic Liquid. *Chem. J. Chinese Universities,* **2017**, 38, 2248–2254.
